# Exploring the Integration Model of Industry Chain Information System Based on Energy Internet and Its Key Technologies

**DOI:** 10.1155/2022/8752048

**Published:** 2022-05-31

**Authors:** Anxun Qi

**Affiliations:** Shuifa Group Co LTD, Jinan 250101, China

## Abstract

Many enterprises have elevated industrial chain management to an important strategic factor for successful operation in a competitive market environment. There are many reasons for the low success rate of enterprise information construction, one of which is that in the process of information construction, only the research of information technology and the construction of information systems are emphasized, but not the integrated management of information resources, and the strategy, mode and method of information resource management are not studied. As a solution for sustainable energy development in the future, the energy Internet has become a hot topic of research in academia and industry today. Integrated innovation, at the enterprise level, is the key to improving core competitiveness and maintaining a sustainable competitive advantage. In order to achieve a deep integration of information technology and energy technology, the energy Internet must be supported by a corresponding system, i.e., an energy information system. In this paper, we propose an information system integration model based on the nature of the industry chain, describe its functional components, and analyze the key technologies of integration. The results show that the integrated model achieves the highest value of each output solution in the time period of 4h, and the calculated transmission capacity of the line is 0.7pu The integrated model of the information system is optimal for the adaptation of the energy environment of each distributed power source in the energy Internet. Therefore, this study provides theoretical support and practical reference for further research on the construction and mechanism of the integrated energy Internet in China's theoretical and business communities, and provides important insights into the construction of the mechanism and countermeasures for the integration of the industrial chain in energy Internet enterprises.

## 1. Introduction

Today's world has shifted from the era of industrial economy to the era of information and knowledge-based service economy [1]. The environment facing modern enterprises has changed dramatically and is still changing [2]. The popular application of electronic data interchange technology, barcode technology, electronic fund transfer technology and Internet/Intranet technology has provided extensive and effective technical support for industrial chain management [3]. Industrial chain management is a systematic, integrated and agile management mode, which integrates new ideas and technologies of modern management today [4]. The modern business environment has prompted enterprises to integrate their resources with external resources to better respond to customer needs, create more value for customers, and create more profits for enterprises [5]. Enterprises use information systems as a tool to develop and utilize a large amount of information resources [6]. Although the development of information technology provides advanced processing means and tools for the development and utilization of enterprise information resources, the effectiveness of its application mainly depends on the mode and method of enterprise information resource management [7].

Therefore, it is important to study the information management system to improve the quality of information management and to ensure the efficiency of the company's management and its ability to handle daily affairs. Information system is a complex network with vulnerability [8]. The vulnerability of an information system is caused by defects in the design and implementation of the hardware and software, related protocols and security strategies that make up the system [9]. When information failures such as interruptions or delays occur in the energy information system, error messages cause the information system to react incorrectly and affect the normal operation of the physical grid [10]. In practice, the variety and scope of information system maintenance operations are expanding, and the number of O&M personnel is increasing, while the diversity and complexity of information systems place higher demands on the professionalism of O&M personnel [11]. Therefore, the energy Internet covering many microgrids can be regarded as a unified energy resource pool, and the orderly flow of energy among microgrids can be controlled through information interaction, which can effectively suppress the limitations of microgrids and ensure the safety and stability of the whole energy Internet.

The successful implementation of industrial chain management cannot be achieved without two enablers, organizational structure and information technology [12]. By adopting industrial chain management, enterprises can improve their market competitiveness [13]. Especially when global industrial chains are formed, the flexibility of enterprises to participate in global competition increases [14]. Industrial chains are not only required to eliminate obvious, inefficient, and non-value-creating activities, but also aim to streamline and intellectualize a series of value-creating activities and eliminate inter-process continuity through the integration of systems [15]. The core of integrated management emphasizes the use of integrated thinking and concepts to guide management practices, which is essentially the integration of elements and complementary advantages. Therefore, it is necessary to strengthen the internal integration and external network of the enterprise, to emphasize internally the parallel development of fully integrated functional departments, to participate in the production of knowledge and information from their respective perspectives, and to research and develop the use of expert systems and simulation models.

The innovation points of this paper are as follows:

1. Based on the existing theories of integrated innovation and energy Internet, the paper defines the concept, connotation and characteristics of enterprise integrated energy Internet from two levels based on the operation mechanism and key technologies of energy Internet, breaking through the previous scholars' definition of the concept of network.
2. Information resource integration as a management practice is one of the core tasks of enterprise informatization, and promoting information resource integration can help eliminate information silos and realize information resource sharing.
3. The integration theory is applied to the study of enterprise energy Internet, and the integration mechanism and model of enterprise integrated energy Internet are constructed from the perspective of integration of goals, information, resources, knowledge, culture and other elements.

The research framework of this paper contains five major parts, which are organized as follows:

The first part of this paper introduces the research background and significance, and then introduces the main work of this paper. The second part introduces the work related to the integration model with the information system of industrial chain, the energy Internet. In the third part, the method of building the functional structure of the industrial chain and the distributed process management model are sorted out, so that the readers of this paper can have a more comprehensive understanding of the method of building the industrial chain information system integration model based on the energy Internet. The fourth part is the core of the thesis, which completes the description of the operation mechanism and key technology analysis of the Energy Internet from two aspects: the operation mechanism and business model analysis and the key technology analysis of the Energy Internet. The last part of the thesis is the summary of the whole work.

## 2. Related Work

### 2.1. Industry Chain Information System Integration Mode

The changes in the environment faced by modern enterprises, especially the development and application of e-commerce with the Internet as the core technology, are triggering a major change that is causing profound changes in the way people live and behave. At the level of the internal chain, management emphasizes the coordination of entities and activities within a single enterprise: the information system is built and implemented mainly using Intranet technology and MRPII/ERP management software. Integrated information systems must require strong compatibility, cross-platform and cross-language operation, and provide reliable access to Web applications. Therefore, enterprises now need to be able to organize, search, and access truly valuable information through the integrated management of information resources.

Yang et al. argue that integrated enterprise information resource management is the process of organizing and integrating information resources in the internal and external environment of an enterprise to support business change and organizational transformation, using the mission, purpose, and strengths of the enterprise as the integration point [16]. Hao and Wang analyze the product development strategy, process, and performance of the pharmaceutical industry in the context of globalization [17]. Sun et al. argue that information resource integration is divided into data integration, content integration, and process integration, and that process integration is based on data integration and content integration [18]. Griffiths proposed that the integration of R&D and marketing activities, as well as the integration of R&D activities and user requirements, has shown an important contribution to NPD under the conditions of technological and market uncertainty [19]. Sun et al. argue that information resource integration includes system integration and sharing, software integration and sharing related to business processes, and information integration and sharing [20].

Therefore, in the face of the challenges and requirements of the new era, it is of great academic value and practical significance to explore the mode and method of enterprise information resource management from the new perspective of integration and to form a set of mode and method of enterprise information resource management to meet the new era background and requirements.

### 2.2. Energy Internet

At present, the contradiction between China's economic development and energy structure is becoming more and more prominent, and the demand for transformation of energy production and consumption patterns is becoming stronger. The information in the Energy Internet covers power supply, grid and load in all aspects, including static basic data describing system parameters, dynamic data monitoring system operation status, and various analysis data. In the context of the energy Internet, the traditional O&M model with equipment as the maintenance object is developing into a new model with users as the service object, which has changed from the current information and communication O&M objectives.

Li et al. introduced the definition, key technologies, and development models of the energy Internet [21]. Users are both consumers and producers.Wang et al. discussed the relationship between the Internet of Things, smart grid, and cloud computing with the energy Internet [22].Ren et al. proposed the technical connotation and technical characteristics of the energy Internet, and analyzed the technical elements and technical forms of the energy Internet [23].Hu et al. Most of the electrical energy in the energy Internet is generated randomly, and thus it is impossible to predict the availability of a certain moment [24]. Yan studied the architecture of the energy Internet and analyzed the key technologies in the process of developing the energy Internet [25]. Stable and reliable power supply is the result of the joint action of all power sources on the whole grid, and these characteristics match well with cloud computing characteristics.

In order to cope with the current severe information and communication operation and maintenance situation and continuously improve the information and communication business level, it is urgent to study a new model of information and communication system operation and maintenance management in the context of the energy Internet. On this basis, we focus on the integration mode of information system of industrial chain based on energy Internet, as well as the physical architecture and technical route of energy information system, and put forward the key technology of energy information system construction.

## 3. Construction Method of Industrial Chain Information System Integration Mode Based on Energy Internet

### 3.1. Method for Construct Functional Structure of Industrial Chain

**Figure 1:**
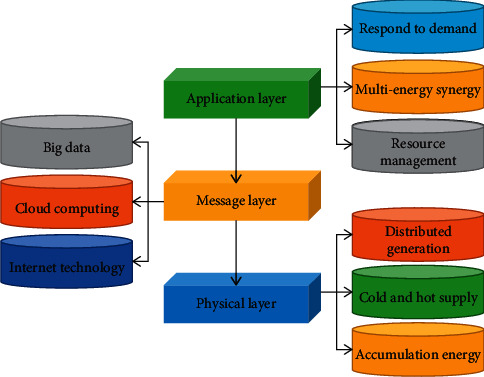
Schematic diagram of energy internet engineering construction architecture.

Business activities of industrial chain management are divided into three levels:strategic level, tactical level and operational level. When conducting inter-enterprise information system integration, it is extremely important to ensure the security of network information in the process of transmission [26]. Energy Internet operational mechanisms and business models are the core of the Energy Internet and are the key to improving comprehensive energy efficiency and building an integrated energy service system [27]. According to the main features of the energy Internet and combined with the Internet technical architecture system, the study proposes a three-layer physical-information-application construction model, which is shown in Figure 1.

First of all, the strategy level is the decision-making level, which is long-term and relatively stable, and determines the general policy, policy and overall planning and design of the enterprise chain, including decisions on positioning, production, inventory, sales and transportation. When a service requester needs to invoke a service it first goes to the service agent to find the service it needs and gets information on how to invoke the service, and then goes to invoke the service published by the service publisher based on this information. Defining the network topology model, the characteristic path length of the network is given by the following equation.

(1) $$\begin{array}{c} L=\sum_{i\neq j}\frac{2*d_{ij}}{\left[N*\left(N-1\right)\right]}, \end{array}$$

*i*, *j*--Any two nodes in the network, *N*--Total number of nodes in the network, *d*_*ij*_--The shortest distance between two nodes

It connects the FMIS system with other subsystems of the MIS and subsystems within the FMIS system into an organic whole by means of data interfaces. The size of the enterprise is determined in the marginal cost of organizing transactions within the enterprise is equal to the marginal cost of organizing the same transactions in the market or in another enterprise [28]. So that they support each other, call each other, have common possession of information and common access to data in order to achieve a holistic effect that cannot be achieved by a single application. The total number of neighboring nodes of a node is called the degree of the node. Looking at the model as a whole, at any point in the network, the degree distribution function of that point is :

(2) $$\begin{array}{c} P\left(k\right)=\frac{2m\left(m+1\right)}{k\left(k+1\right)\left(k+2\right)}\propto 2m^{2}k^{-3}, \end{array}$$

*m*--Number of adjacent nodes, k--Parameters for calculating degrees

**Figure 2:**
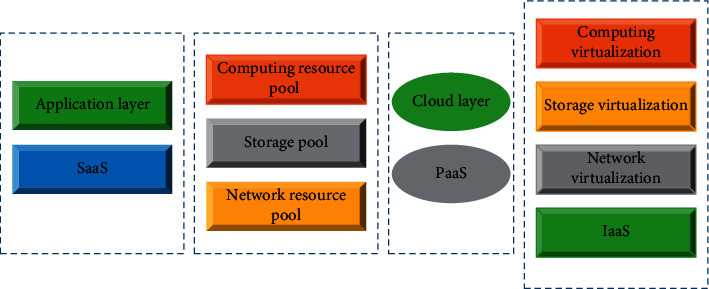
Functional structure of industrial chain.

Therefore, it is necessary to streamline the scale of enterprises, reduce the degree of integration of enterprises, and externalize certain inefficient departments and functions from within the enterprise, leaving only the core functions of the enterprise with the most competitive advantage. The functional structure of the industrial chain is shown in Figure 2 below.

Secondly, the tactical and operational levels are execution levels that develop short- and medium-term plans and are characterized by short and dynamic role cycles. Service agents give services the mechanism to publish (register and classify) themselves and their services, and also provide service requests with the mechanism to find the services they need [29]. The firm or cluster of firms that is in the position of an innovation agent is called a core firm, and a vertical network is formed between the core firm and the suppliers of upstream and downstream relationships as well as users. The aggregation coefficient of nodes is defined as the ratio of the number of edges E_i_ that actually exist between these k_i_ nodes to the maximum number of edges that theoretically exist, as.

(3) $$\begin{array}{c} C_{i}=\frac{2E_{i}}{k_{i}\left(k_{i}-1\right)}. \end{array}$$

Further, the aggregation factor of the network can be defined.

(4) $$\begin{array}{c} C=\frac{1}{N}\sum_{i=1}^{N}C_{i}. \end{array}$$

According to the role mechanism of enterprise information resources, combined with the distribution, status and demand of information resources in the enterprise, we can generally consider enterprise information resources integration from three aspects: the goal of enterprise information resources integration, the content of integration and the scale of integration. Application Integration (EAI) middleware is deployed in each department and data center, and departmental data exchange platform and central data exchange platform are established respectively to realize data exchange and sharing among departments. To improve the information communication network within the system, realize the two-way flow and effective utilization of electrical information data within the power grid, and on this basis, realize the effective control of the power grid dispatching center on the distribution network. For each virtual node, *j* is a certain neighboring node that is making a decision. The transmission probability based on dynamic information is defined as

(5) $$\begin{array}{c} P_{i,j}=\frac{e^{-t_{j}}}{\sum_{l\in N\left(i\right)}e^{-t_{l}}}. \end{array}$$

Finally, the operational level is related to daily business activities such as inventory management, production activities, equipment management, specific job scheduling, etc. In the process of information resource integration, enterprises must determine the objectives of integration according to their specific status and needs, so that the integration can be targeted and the role and potential of enterprise information resources can be fully and effectively played. Through rapid data exchange, real-time integration of data from relevant departmental business systems or interaction between business systems, so that leaders and departments can make comprehensive use of the results of information integration to assist in decision-making and management.

### 3.2. Distributed Process Management Model

**Figure 3:**
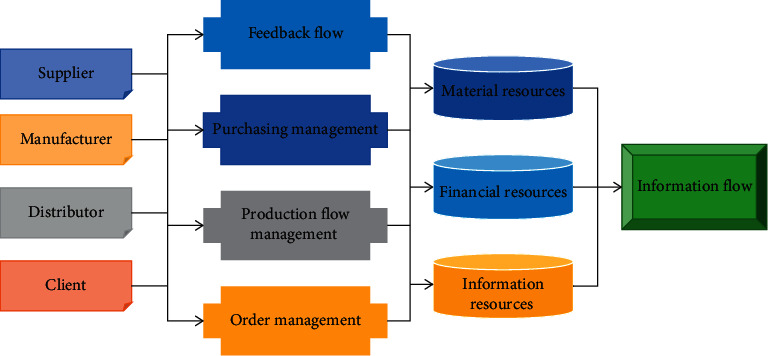
Distributed process management model.

The industrial chain system is based on the synergy and information sharing among multiple enterprises [30]. The industrial chain is not static, and as the market environment changes, the members in the chain will also change, with new members joining in and old members possibly dropping out. According to the main features of the energy Internet, combined with the Internet technology architecture system, the study proposes a three-layer construction model of physical-information-application. Because of this, many companies have elevated industry chain management to an important strategic factor for successful operation in a competitive market environment. The distributed process management model is shown in Figure 3 below.

First, the model includes interfaces for interoperability and communication between components in five categories. The physical layer focuses on addressing multiple energy sources interconnection and grid vertical intelligent interaction. The industry can design and build a dedicated network system according to their actual needs to best meet their needs. The diversity of information formats is manifested in structured data of business systems, semi-structured data of network systems and unstructured data of system log files. The distributed generation and exchange power from the main grid must be equal to the energy LAN consumption power, so the power balance equation is shown below：

(6) $$\begin{array}{c} \sum_{i}^{NDG}P_{i}\left(t\right)+P_{\text{Grid}}\left(t\right)=P_{\text{Load}}\left(t\right)+\sum_{n=1}^{NPHEV}P_{\text{PHEV}}\left(t\right). \end{array}$$

*P*_Load_(*t*)--Load power, *P*_*PHEV*_(*t*)--Charge/discharge power

The global data that needs to be transmitted, exchanged, processed and shared throughout the MIS and among various subsystems for the purpose of information integration of commercial banks are mainly the mainframe business transaction data of the enterprise. Both the degree of node and the waiting time of stream have an impact on the routing efficiency of the system. Therefore, the weights of node degree and waiting time for calculating the probability of selecting adjacent nodes for transmission are adjusted by adjusting this parameter in the following way：

(7) $$\begin{array}{c} P_{i,j}=\frac{k_{j}^{\alpha }e^{-\left(1-\alpha \right)t_{j}}}{\sum_{l\in N\left(i\right)}k_{l}^{\alpha }e^{-\left(1-\alpha \right)t_{l}}}. \end{array}$$

Second, the process definition tool interface, client interface, call application interface, enabling component interoperability interface, management and monitoring program interface. The information layer applies big data cloud platform technology to address the interconnection of massive information from multiple sources. The information can be divided into 3 categories according to the role of static information such as grid equipment parameters and topology, business information such as power generation and load demand changes and transactions, and dynamic control information for grid operation and monitoring. By using Internet network for integration, enterprises can build an integrated industry chain information system with good scalability and interoperability (most network systems are based on TCP/IP protocol) without building their own private networks or paying expensive leased line fees. The Bessel formula is then used to calculate the standard error of the original data and replace it with the replacement value, and the formula for calculating the width of the replacement value code is:

(8) $$\begin{array}{c} B=\frac{V}{\left(F\times 2^{\xi }\right)}. \end{array}$$

*V*--Optional range of data acquisition, *F*--Optional gain of data acquisition, *ξ*--Resolution of collector

The clustering formula is as follows The clustering formula is:

(9) $$\begin{array}{c} E=\sum_{j=1}^{K}\sum_{x\in C}{\left|xj-\bar{xj}\right|}^{2}. \end{array}$$

*E*--Information clustering function, *x*_*j*_--Data set, $\bar{x_{j}}$--Mean data set, *C*--Cluster center of data set, *K*--Cluster collection

Local data is the data exchanged and shared within each subsystem of the MIS system, and transferred, exchanged and manipulated within the subsystem. It inherits and develops the advantages of various systems such as design resource management, design process management and information management, and applies parallel engineering methodology, network technology, database and object-oriented technology. For network actors, the network provides various formal and informal communication channels, thus promoting the flow of knowledge, which is very important for the dissemination of new ideas and innovation. In order to simplify the operations in the application calculations, the output power can be calculated by the following equation：

(10) $$\begin{array}{c} P_{pv}=P_{STC}\frac{G\left(T\right)}{G_{STC}}\left(1+k{\left(T_{c}-T\right)}_{r}\right). \end{array}$$

Finally, each interface develops a series of APIs to standardize the function invocation and data exchange formats between the two sides of the component. The application layer focuses on solving the new energy production and consumption, market and trading model based on Internetization. In addition to having the traditional grid operation and management data, the energy Internet, due to the access of new energy sources, for wind and photovoltaic energy sources, must be based on a large amount of meteorological background data for the prediction of electrical energy output. Develop a software architecture that integrates the various information and application resources involved in the business process of an enterprise into one information system, so that different information and applications can be shared and interacted with each other and operate as an integrated whole. In the process of data management, each subsystem handles and processes private data to produce local data, and through the exchange and processing of local data, global data is generated within the subsystem for delivery and exchange to the whole system. Its emergence provides a new solution to the problem of enterprise information integration.

## 4. Analysis of Operation Mechanism and Key Technologies Based on Energy Internet

### 4.1. Analysis of Operation Mechanism and Business Model

The industrial chain is a concept containing four dimensions: value chain, enterprise chain, supply and demand chain and spatial chain. It is an up-and-down and dynamic chain intermediate organization formed by enterprises in the same industry or different industries with the goal of satisfying users' demands based on specific logical links and spatial and temporal layouts. A suitable operation mechanism and business model can provide energy enterprises, power sales companies, users, owners of distributed resources and other types of subjects to participate in the implementation path of energy Internet operation and create an open energy Internet ecology.

**Figure 4:**
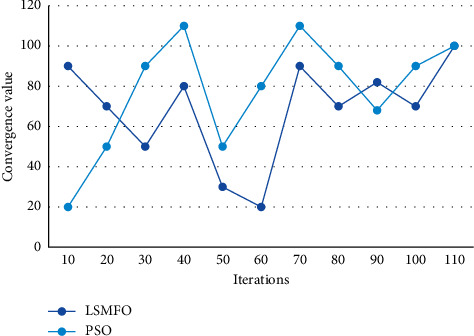
Convergence characteristics of two algorithms.

First, the price-based multi-energy cooperative operation mechanism forms a method for optimizing the operation of cross-region multi-energy systems at the city level. In order to accurately reflect the characteristics of the regional integrated energy network, the network of energy production, transmission and use constituted by multiple energy forms needs to be properly modeled. The characteristics of intermittency, instability and small scale affect the safe and stable operation of the microgrid. the solution process of 2 intelligent optimization algorithms for energy internet energy economic adaptation optimal scheduling is shown in Figure 4.

**Figure 5:**
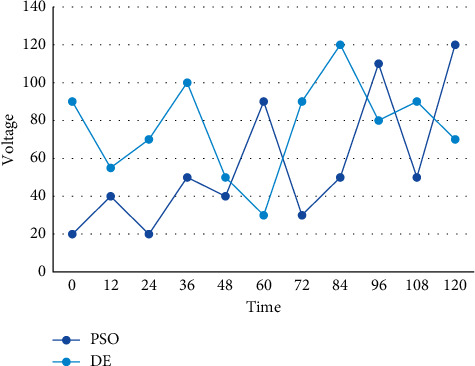
Comparison of a set of solutions of DE and PSO on battery.

Compared with PSO, LSMFO has a faster convergence rate in the early stage and a weaker search capability in the later stage, while the optimization performance presented at the end is also less than ideal. The deep integration of multi-party industry chain is a prerequisite for the establishment and normal operation of energy Internet enterprises. The information and communication operation and maintenance services are controlled in real time to improve service quality and ensure safe and reliable business. The weak link of the whole energy information system is no longer limited to the internal physical system, and the failure of the information link will become more common and diverse, which will most likely further endanger the energy system. The system completes the cloudization of information network and hardware resources, and provides computing capability, storage capability, data analysis capability, and application integration capability for the application system layer, etc. The distribution of DE and PSO in the energy Internet energy economic adaptation regarding battery decoupling is shown in Figure 5.

**Figure 6:**
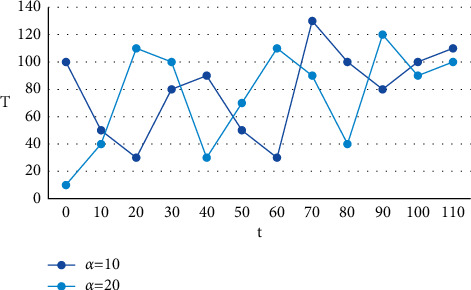
T and t with different *α* values.

Secondly, the virtual power plant day-ahead scheduling model based on double-layer planning can realize dynamic prediction of distributed new energy generation capacity in a specific region. The establishment and development of energy Internet enterprises must break the barriers between industrial chains and achieve the deep integration of all industrial chains. The real-time dynamic characteristics of information are mainly reflected in two aspects: first, the ability to obtain system operation status data and user demand information in real time; second, the ability to realize high-performance computing of massive data. The connection between various enterprises in the industrial chain needs to be closer, and the resource allocation in all aspects needs to be more flexible and reasonable. In the Internet local routing mechanism, the algorithm based on dynamic information is calculated based on the length of the node's own data queue and the queue length of neighboring nodes, and the T and t pairs under different *α* values are shown in Figure 6 below.

To carry out hierarchical management of the generation and maintenance enterprises, determine a pool of competent and trustworthy generation and maintenance enterprises, and establish a long-term cooperation mechanism. The enterprises in the chain are divided into basic and auxiliary enterprise members, so as to further analyze the key processes and auxiliary processes in the chain and their characteristics. After the power layer converter gets the command value, it starts to adjust the power flow, and the results are shown in Table 1.

Finally, drawing on the Internet thinking model, an open integrated energy operation service platform should be built to provide data capabilities and business capabilities for each subject to carry out integrated energy operations. It should also model the information network composed of multiple communication technologies and media, reflecting the mutual influence and interaction between the energy network and the information network. It is to model the value chain system of the industry chain, determine the starting position of the industry chain, describe the role and role of enterprises in the industry chain, and analyze the problems and their root causes in the value system of the industry chain. Due to the development of enterprises in many aspects such as massive renewable energy access, large-scale energy storage and information technology demand interaction, a large amount of data information from multiple sources such as energy production, energy storage information and user consumption characteristics will be generated. Therefore, it is necessary to complete the control of information and communication operation and maintenance from the organizational structure, and establish an internal management organization that can fit the scope of operation and maintenance business and adapt to market changes. The increasing integration of information link and energy system is no longer a simple information flow regulating energy flow, but a real integration of information flow and energy flow.

### 4.2. Analysis of Key Technologies Based on Energy Internet

**Figure 7:**
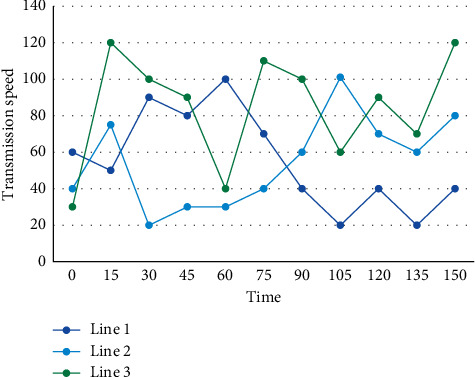
Data transmission rate of communication line.

The traditional work-based organizational approach and organizational management model, with its emphasis on work as the core and individuals as subordinate to work, has neglected the critical resources and important components of organizational competitiveness of today's organizations. Complex network theory provides another theoretical basis for mechanism-based modeling and simulation of intercontinental energy networks. The data transmission rate of the communication line is shown in Figure 7.

In order to realize the prerequisites provided by the integration of information and energy systems to the construction and operation of energy Internet enterprises, and to truly realize the linkage of supply and demand among multiple subjects, it is necessary to break the boundary restrictions between different industries and strengthen the information security of all parties. The energy Internet-based industry chain information system aims to build a convenient and efficient information collection, processing, analysis and display platform with the high concurrency, high reliability, high scalability and low cost of cloud computing. Its key technology is aimed at supporting the physical energy grid to achieve integrated energy interconnection, efficient and flexible distribution network, and intelligent and efficient network.

**Figure 8:**
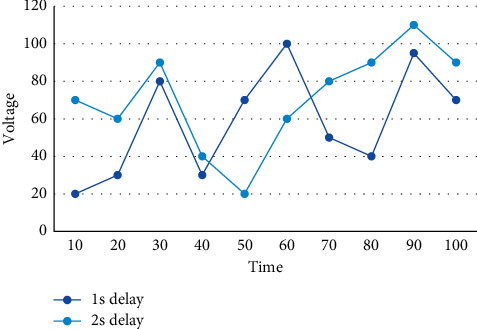
A schematic diagram of the voltage dynamic change of node 600 under different communication delay conditions.

Firstly, the third generation semiconductor Si C is used to form a wide forbidden band power device, which has lower switching loss and lower on-state voltage drop to enhance the conversion efficiency of the device. Since the power is generated centrally and the grid is dispatched and managed in a unified manner, the safety and stability of the system can be ensured by realizing effective dispatch management of the grid. The processing of these public data is realized through the functional modules of FMIS system to realize the processing, transmission and sharing of data among the functional modules. Therefore, it is not the management of each specific database by PDM, but the management of information integration on a macro level, the rise of local automation to overall automation, and the transformation of local optimum to overall optimum. Under ideal communication conditions, there is no delay in data transmission, and the node voltage can immediately respond to load changes, while with the communication delay, Figure 8 is a schematic diagram of the dynamic change of voltage at node 600 under different communication delay conditions.

Secondly, the multi-port DC circuit breaker topology with shared transfer branches and the development and application of three-port DC circuit breakers greatly reduces the number of electronic devices and reduces the volume and cost. The Energy Internet requires data centers to provide richer information services and a high-performance distributed computing environment for data mining and assisted decision-making. It integrates the right business data, business specifications, business procedures, policies and controls. Security services to control access between industry chain member companies, information is passed between industry chain member companies, security is a key issue that needs to be addressed and must ensure that information is only available to the appropriate members in an accurate manner. the openness of the PDM kernel is mainly reflected in the system object-oriented modeling technology to build the system management model and information model, and provide object management mechanisms to achieve product information Management Table 2 shows a set of optimal results of the integrated model for energy adaptation and scheduling of each distributed power source in the energy Internet based on environmental costs.

When the time period is 4h, each output solution reaches the highest value, and the transmission capacity of the line is calculated to be 0.7pu The integration mode of the information system is optimal for the energy environment adaptation of each distributed power source in the energy Internet.

Finally, the engineering and technology scheme of control and protection integration and compact design is proposed to realize the functions of coordinated control of multi-terminal flexible DC distribution network, one-key system start-stop, and seamless switching of multiple operation modes. It can combine the business processes and business logic of an enterprise to provide predetermined functions to assemble the application system that suits the needs of the enterprise. The key used for encryption is different from the key used for decryption, and the decryption key cannot be calculated from the encryption key. The responsibility of the service provider is to invoke its own information system according to the w SDL document provided by the service proxy center, implement the corresponding functions, and register and publish them in the proxy center so that they can be accessed by other applications, accept and handle calls from Web service requestors.

## 5. Conclusions

Enterprise informatization is the process of reshaping enterprise competitiveness through informatization, in which the integrated management of information resources is the key to enterprise informatization. The security framework of integrated industrial chain information system is a topic between the industrial chain field and the network security field, which is of great relevance today when the network security issue is of great concern. The energy Internet is more complex than the smart grid currently being carried out, and places higher demands on network transmission performance, data storage management, data analysis and mining, and information interaction. From the depth of integration and utilization of enterprise information resources, it will gradually develop from information integration to knowledge integration, and then to human integration, so as to realize the “human-centered” management idea in the enterprise. According to the direction and interrelationship of enterprise integration, the integration methods of enterprise energy Internet can be summarized as industry-production network, industry-academia-research network, government-industry-academia-research network and its government-industry-academia-research-gold network. Carrying out research on the theoretical basis and practical path of the energy Internet and realizing the interconnection of energy resources are of strategic importance to China's future energy security. Therefore, this paper proposes an industrial chain information system integration model based on the energy Internet, starting from the research framework of enterprise information system integration, from the perspective of the overall construction of the entire industrial chain management system, and analyzing its characteristics and key technologies. The energy Internet and the industrial chain interact with each other, and jointly promote the interactive integration of enterprise integrated energy Internet, and make the network produce synergistic operation effect, information sharing effect, common vision effect, complementary capability effect and knowledge transfer effect, and promote the development of cooperative relationship among members of integrated energy Internet.

## Figures and Tables

**Table 1:** Active current distribution of each line after using the power routing strategy.

	Active power/Pu	Cost
Line parameters	0.5	6
MAS calculated value	0.4	3
Active control value	0.7	9

**Table 2:** Output solutions of integrated mode in distributed generation of energy Internet.

Time period (h)	1	2	3	4
Battery (power)	-25	-18	14	28
Fuel cell (power)	3.672	4.663	5.763	6.871
Microcomputer (power)	8.54	9.65	10.26	11.76

## Data Availability

The dataset can be accessed upon request. The data used to support the findings of this study are available from the corresponding author upon request.
